# Late Migration of a Transcatheter Heart Valve

**DOI:** 10.1016/j.jaccas.2023.102214

**Published:** 2024-01-05

**Authors:** Zaid Alirhayim, Dean Kereiakes, Santiago Garcia

**Affiliations:** Christ Hospital Physicians, Cincinnati, Ohio, USA

**Keywords:** aortic stenosis, device migration, TAVR

## Abstract

Migration or embolization of transcatheter aortic valve replacement (TAVR) prosthesis is an uncommon (usually acute) complication. Associated risk factors include malpositioning, pacing miscapture, undersizing, postdilation, and bicuspid anatomy. Delayed migration is exceedingly rare with little experience reported. The presentation can be catastrophic, requiring emergency surgery. Herein, we present a case of late TAVR migration managed with repeat transfemoral TAVR.

## Graphical abstract

**Figure:**
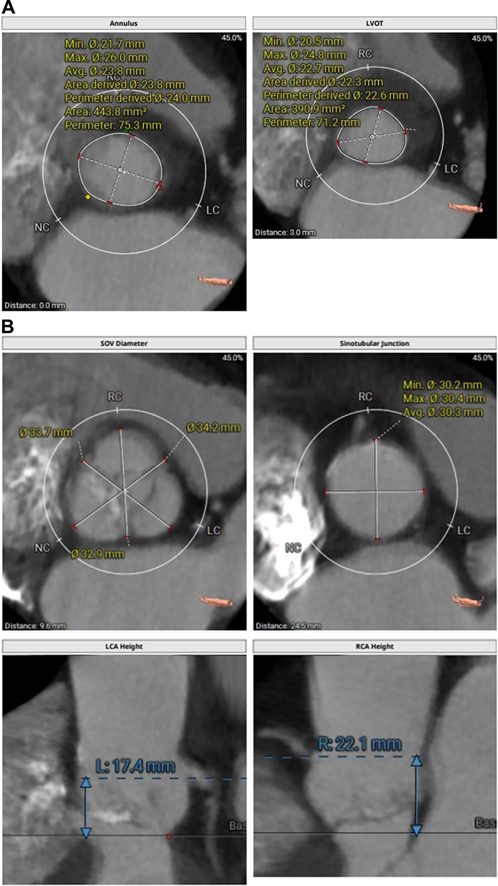

## History of Presentation

An 80-year-old man with a history of transcatheter aortic valve replacement (TAVR) presented to our structural heart disease clinic for a scheduled visit. One year prior, he had undergone TAVR at our institution with a 23-mm S3. The postprocedure course was significant for persistent conduction disturbances, which required implantation of a dual chamber pacemaker. The patient did well otherwise. He was asymptomatic and physical examination was unremarkable. Routine echocardiogram now demonstrated a mean aortic gradient of 27 mm Hg (increased from 7 mm Hg, 1 month after the index procedure).

**Learning Objectives**

• To formulate a differential diagnosis in the patient with elevated gradients after TAVR.
• To understand the technical considerations of redo-TAVR in the stable patient with migrated TAVR prosthesis.

## Past Medical History

The past medical history included hypertension, prior transient-ischemic-attack, and coronary artery disease with previous percutaneous transluminal coronary angioplasty to a large diagonal branch.

## Differential Diagnosis

Our main differential diagnoses were hypoattenuated leaflet thickening and early structural valve deterioration. Patient–prosthesis mismatch was felt to be unlikely because the postprocedure gradients had been normal.

## Investigations

**Figure 1:**
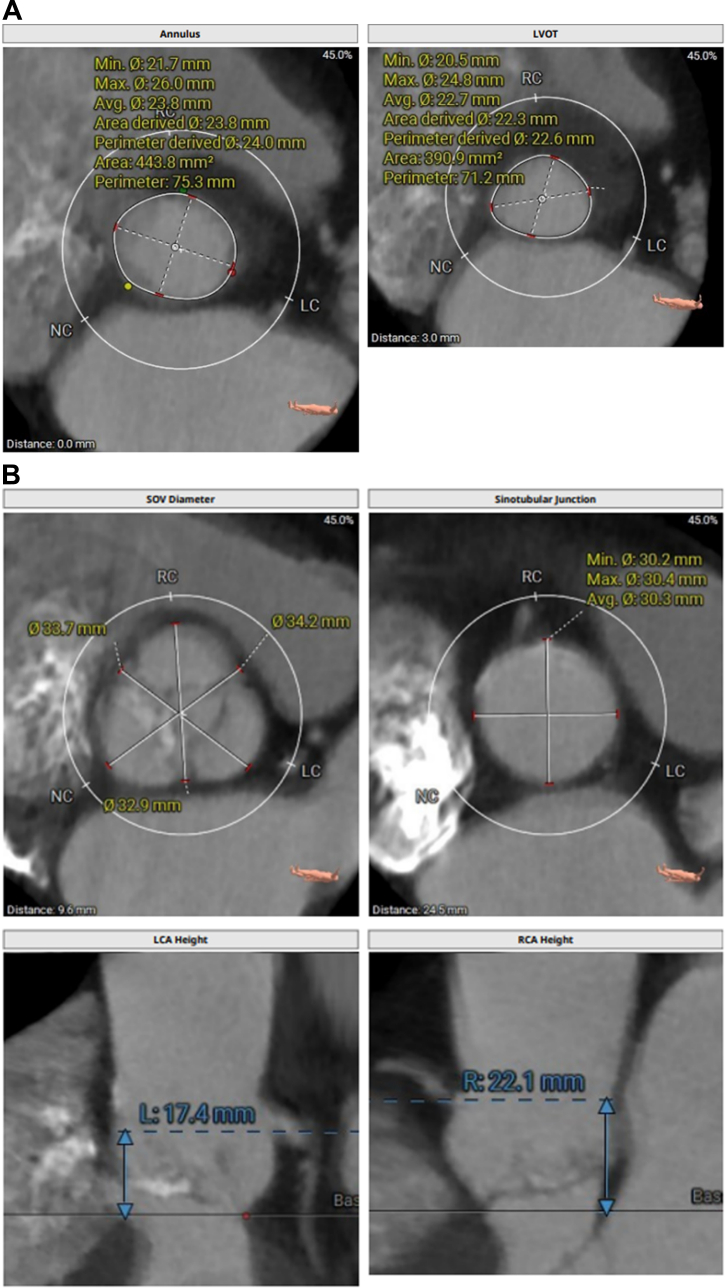
CTA Images (A and B) Index CTA images with annular and LVOT measurements. CTA = computed tomography angiography; LC = left coronary cusp; LVOT = left ventricular outflow tract; NC = non coronary cusp; RC = right coronary cusp.

**Figure 2:**
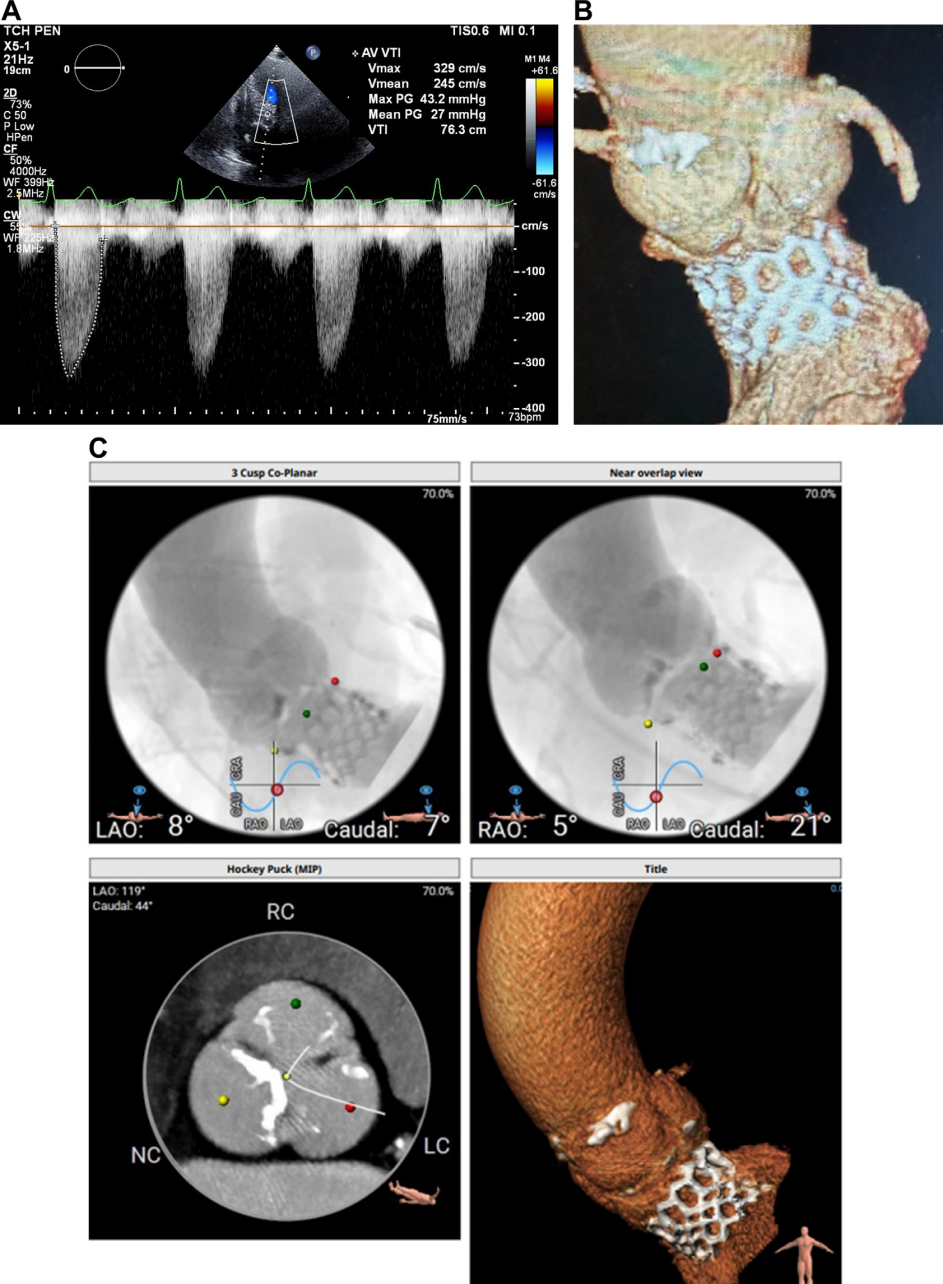

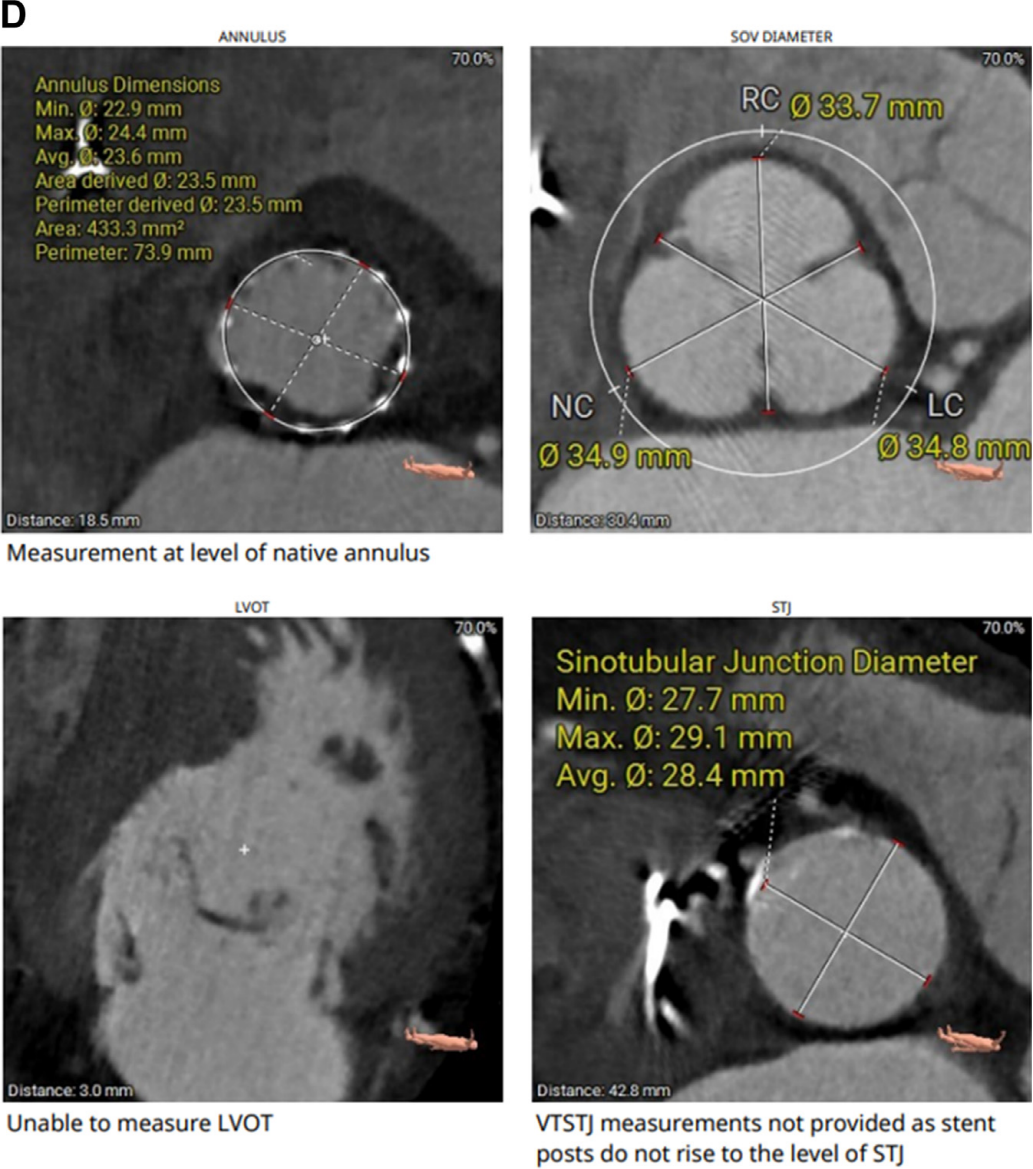

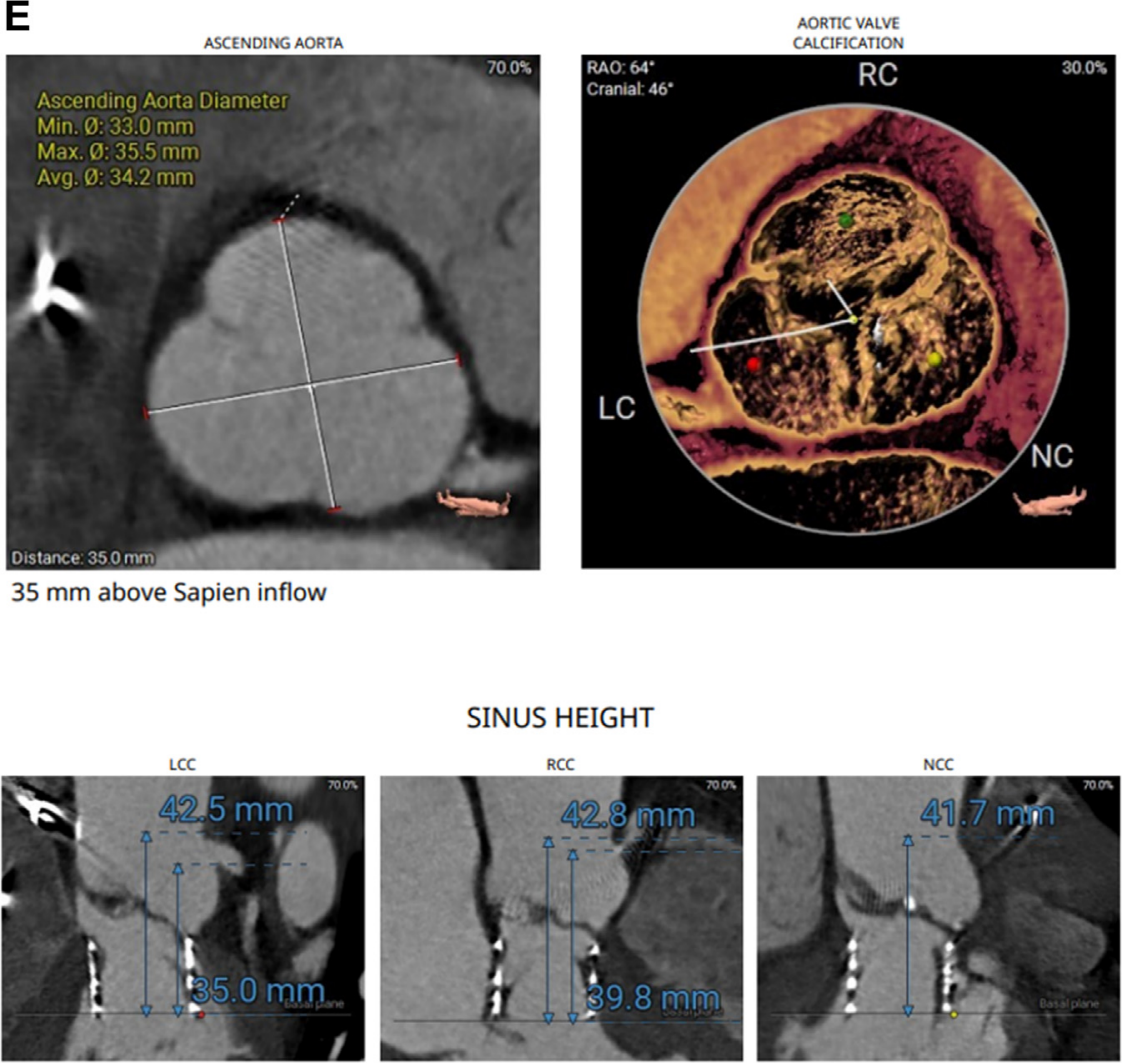

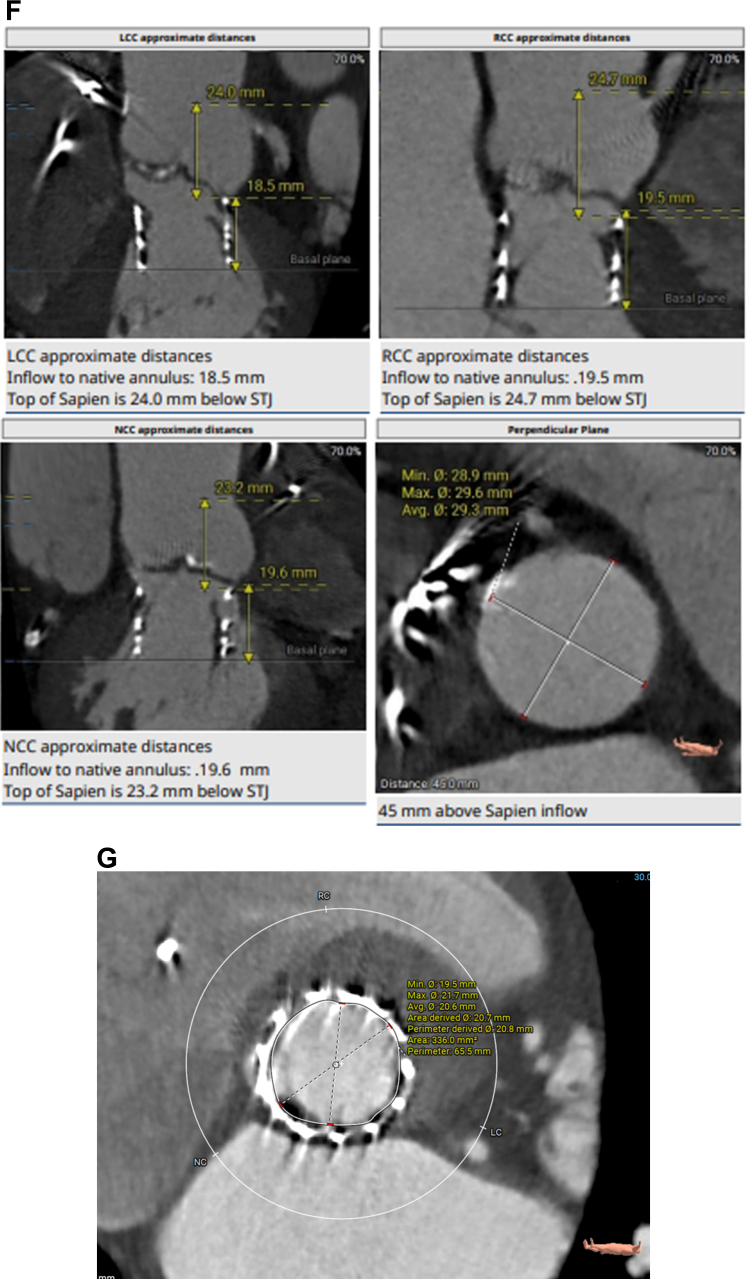
CTA images (A) CW Doppler echocardiogram showing increased gradients across the aortic valve. (B to G) Repeat CTA images after S3 migration into the LVOT below the aortic valve annulus. Abbreviations as in Figure 1.

His echocardiogram showed normal left ventricular ejection fraction, restricted native aortic valve opening with a mean gradient of 27 mm Hg, and a more ventricular position of the TAVR prosthesis (Video 1). Review of the index computed tomography angiography (Figures 1A and 1B) showed an annular area of 442 mm^2^, at the margin between 23 and 26 mm when sized for an S3 valve. Given the smaller left ventricular outflow tract (LVOT) dimensions, a 23-mm S3 had been selected. Notably, index imaging had shown only mild leaflet calcification, without LVOT or annular calcification. The initial cine-angiogram (Video 2) demonstrated deployment in a 70/30 position, with 70% of the stent frame in the aortic position and 30% in the LVOT. There had been moderate paravalvular regurgitation (PVL) (Video 3), which immediately resolved after postdilation (Video 4) with an additional 3 mL of volume in the inflation device. Because of the echocardiographic findings (Figure 2A), we proceeded with a repeat computed tomography angiography (Figures 2B to 2G), which revealed complete retrograde migration of the S3 into the LVOT, with its outflow located 1.5 mm below the native aortic annular plane. There was no evidence of hypoattenuated leaflet thickening or structural valve deterioration.

## Management

**Figure 3:**
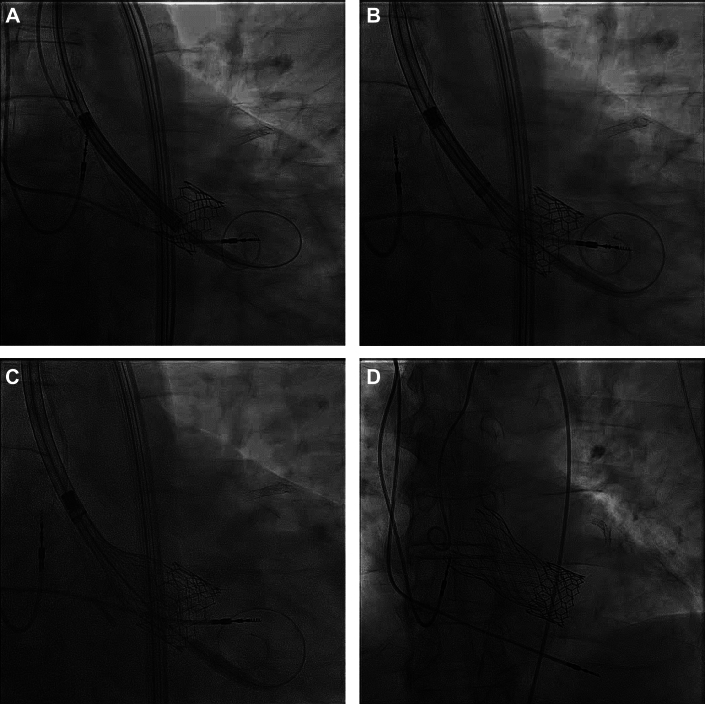
Redo TAVR (A to D) Redo TAVR, deployment steps in sequence. TAVR = transcatheter aortic valve replacement.

The patient was reviewed by the multidisciplinary heart team for consideration of surgical explant of the TAVR prosthesis with SAVR, versus redo TAVR. Given the patient’s advanced age, lack of significant interaction with the aortic-mitral curtain by the displaced transcatheter heart valve (THV) (mean mitral gradient, 4 mm Hg), and increased surgical risk, a decision was made to proceed with redo TAVR. The goal of the procedure was to treat the native valve AS and to prevent further migration of the original prosthesis. Owing to concerns regarding acute ventricular embolization of the TAVR prosthesis during the procedure, our cardiothoracic surgeons prepared a bail-out surgical plan with a primed cardiopulmonary bypass circuit on pump standby. We crossed the aortic valve with an AL1 diagnostic catheter and a straight guidewire. Proper positioning of the guidewire through the orifice of the THV was confirmed by rotational angiography (Videos 5A and 5B). The AL1 was exchanged for a pigtail catheter through which a curved pre-shaped Circulo 0.035-inch wire (Abbott Vascular) was placed at the LV apex. Balloon predilatation was not performed. A 26-mm Evolut FX valve was overlapped with the frame of the previously implanted S3 (Figures 3A to 3D). Valve function was confirmed by root angiography and echocardiography (Videos 5C and 5D). The THV delivery system was withdrawn and partial reversal of heparin was achieved with 20 mg of protamine sulfate. The patient was monitored in the stepdown unit and discharged home the next day.

## Follow-up

Four months later, the patient remains asymptomatic, with stable position of the valves on fluoroscopy. Recent echocardiography shows a mitral gradient of 6 mm Hg across the aortic valve without PVL.

## Discussion

Migration of TAVR prostheses is a rare complication that typically occurs during the intraprocedural setting or on the first day afterward. Late migration months or years later is exceedingly uncommon, with only a handful of previously described case reports.^1^, ^2^, ^3^, ^4^, ^5^, ^6^ Table 1 lists some of the potential risk factors for acute or delayed THV migration. Theoretically, migration of TAVR valves (whether acute or delayed) can occur when the valve is placed too low in the aortic annulus, resulting in less anchoring of the THV to the leaflets, which creates leaflet overhang that exerts a continual downward force on the valve.^6^ The presence of asymmetric calcification or bicuspid aortic anatomy can also be potential contributors, although neither factor played a role in our patient. We believe that undersizing in the setting of minimal leaflet or annular calcification probably contributed to delayed TAVR migration in our patient. What role postdilation or stent frame recoil played in this case is unknown. In retrospect, the capacious sinus dimensions would have accommodated a larger self-expanding valve option, such as a 27-mm Navitor (Abbott) or a 29-mm Evolut (Medtronic). Either choice may have been more appropriate than the 23-mm S3 used in the index procedure, especially in a male patient. All things being equal, one may consider an alternative TAVR platform when in between sizing parameters on a specific valve platform. The next generation SAPIEN X4 (Edwards Lifesciences) contains features that might help to address some of the challenges of sizing THV when the aortic annulus falls between 2 valve sizes. The reconfigured stent frame is designed to reduce foreshortening and facilitate tailored sizing with expansion across a wide range of diameters at 0.5-mm increments. Therefore, 3 valves sizes (23 mm, 26 mm, and 29 mm) allow 16 deployment diameters (21.5-30.0 mm), which could favorably impact paravalvular regurgitation and mitigate the risk of late valve migration. The ALLIANCE trial (Safety and Effectiveness of Balloon-Expandable Bioprosthetic SAPIEN X4 Transcatheter Heart Valve) is investigating the safety and efficacy of this new iteration of the SAPIEN valve.

**Table 1:** Factors That May Contribute to Retrograde TAVR Migration

Undersizing Suboptimal valve expansion Prosthesis malpositioning (low implantation) Functional or anatomical bicuspid valve Postdilation of the valve Uneven or insufficient aortic annulus calcification leading to inadequate prosthesis fixation, Aortic paravalvular regurgitation

TAVR = transcatheter aortic valve replacement.

Although a migrated THV can present catastrophically with myocardial ischemia (owing to obstructed coronary ostia), acute HF, and shock (owing to conduction abnormalities, acute aortic regurgitation or sudden embolization), this case shows that the presentation can be more subtle, isolated to increased valve gradients.

The management of delayed THV migration should consider the clinical condition of the patient, their anatomical suitability for a transcatheter option (including whether the migrated valve is impinging on the mitral apparatus), and the associated risks of each approach. In our literature review of late TAVR migration, most patients underwent surgical management. In 1 instance, retrograde migration into the LVOT occurred 2 years after a valve-in-valve TAVR with a balloon-expandable valve, with the patient presenting in cardiogenic shock owing to severe paravalvular aortic regurgitation requiring surgical intervention.^1^ In yet another case of delayed retrograde migration to the LVOT, the patient underwent redo TAVR attempt 6 weeks after initial valve implantation, but because the second THV crossed the first implanted valve, “it fell into the left ventricle,” which warranted immediate surgical intervention.^5^ As an alternative to surgery, some investigators have suggested that a transapical approach in this setting may be safer,^4^^,^^5^ arguing that the transapical approach can mitigate the risks of retrograde wire crossing of both native and migrated valve with potential further dislocation. Because our patient was hemodynamically stable, we felt that a redo TAVR option through the transfemoral approach was feasible with careful and meticulous technique. We also felt that, with careful planning, a surgical approach could still be performed if redo TAVR failed, with advanced priming of the cardiopulmonary bypass circuit and the presence of a surgical team in the hybrid operating room.

Several procedural aspects warrant mention. First, as an added precaution before advancement of the second THV, we would recommend performing rotational cineangiography to confirm that the guidewire has crossed through the opening of the previously implanted valve and not behind its stent frame. The choice of whether to use a self-expanding or balloon-expandable valve should be informed by computed tomography planning, weighing the risks of landing lower or higher than intended, with resultant aortic regurgitation through the second THV skirt at the aortic annulus level. Although either option may have been possible, we selected a self-expanding valve instead of a balloon-expandable valve (BEV). If we had used another BEV for the redo TAVR, we would have likely selected a second 23-mm S3 to overlap with the first one, which had an average diameter of 21 mm when measured inside the metal frame at the outflow. We were concerned that the intra-annular design of the BEV would pose a challenge, particularly relating to elevated THV gradients after the procedure. We, therefore, selected a self-expanding valve for the redo TAVR with the goal of overlapping with the S3 frame and pinning its leaflets, while allowing us to achieve lower gradients. In patients who are unstable, those with a low surgical risk, or those with significant interaction with the mitral valve leaflets, a surgical approach is likely preferable.

## Conclusions

Delayed TAVR prosthesis migration is an uncommon clinical scenario. Its management is often risky and complex, usually requiring surgery. Redo TAVR is a feasible option in selected cases, subject to careful planning and a thoughtful approach.

## Funding Support and Author Disclosures

Drs Garcia and Kereiakes has received institutional research grants from the Lindner Center as well as industry support from Edwards Lifesciences. Dr Alirhayim has reported that he has no relationships relevant to the contents of this paper to disclose.

## Appendix

Video 1 Echocardiogram demonstrating ventricular migration of indexTAVR prosthesis.

Video 2 Deployment cine-angiogram of the S3 valve.

Video 3 Moderate PVL demonstrated after the initial S3 deployment.

Video 4 Aortogram after +3 mL postdilatation of the S3 valve, demonstrating resolution of paravalvular regurgitation.

Video 5A (A) Initial aortogram during redo TAVR procedure. (B) Rotational cine-angiogram confirming passage of the guidewire through the transcatheter heart valve orifice and not behind the stent frame. (C) Aortogram after redo-TAVR procedure, demonstrating stable valve position and without paravalvular regurgitation. (D) Echocardiogram after redo TAVR.

Video 5B (A) Initial aortogram during redo TAVR procedure. (B) Rotational cine-angiogram confirming passage of the guidewire through the transcatheter heart valve orifice and not behind the stent frame. (C) Aortogram after redo-TAVR procedure, demonstrating stable valve position and without paravalvular regurgitation. (D) Echocardiogram after redo TAVR.

Video 5C (A) Initial aortogram during redo TAVR procedure. (B) Rotational cine-angiogram confirming passage of the guidewire through the transcatheter heart valve orifice and not behind the stent frame. (C) Aortogram after redo-TAVR procedure, demonstrating stable valve position and without paravalvular regurgitation. (D) Echocardiogram after redo TAVR.

Video 5D (A) Initial aortogram during redo TAVR procedure. (B) Rotational cine-angiogram confirming passage of the guidewire through the transcatheter heart valve orifice and not behind the stent frame. (C) Aortogram after redo-TAVR procedure, demonstrating stable valve position and without paravalvular regurgitation. (D) Echocardiogram after redo TAVR.
